# Preview position versus length: Key factors in the time course of parallel processing in multitasking

**DOI:** 10.3758/s13421-025-01780-3

**Published:** 2025-09-24

**Authors:** Jovita Brüning

**Affiliations:** 1 Otto-von-Guericke Universität Magdeburg, Magdeburg, Germany; 2 Technische Universität Berlin, Berlin, Germany

**Keywords:** Multitasking, Task switching, Inter-individual differences, Parallel processing

## Abstract

Multitasking research has shown that individuals differ in whether they prefer a more serial or a more parallel mode of task processing at the level of whole tasks. Such preferences can be identified using the task switching with preview (TSWP) paradigm. This paradigm allows, but does not require, individuals to preview the stimulus of the next task switch in a predictable task switching procedure [AAABBB...]. Although several studies have shown that some participants consistently use the preview information, it is still unclear when exactly this information is used and, thus, how parallel processing takes place. The present study is an important step in clarifying this issue. In two experiments, I investigated when exactly individuals who prefer parallel processing during task switching use a preview to prepare for the next task switch. In Experiment 1, the onset and thus the length of the preview was varied within participants. This allowed to disentangle whether parallel processing of the preview depends on the length of the preview (i.e., its likelihood increases with longer preview presentation), or occurs contingent on a single trial (i.e., is a rapid process). Strikingly, parallel processing occurred regardless of preview duration, suggesting that a short preview may be sufficient. In Experiment 2, participants received the preview in discrete steps, i.e., with the same length throughout the sequence. There was a clear peak in the use of the preview immediately before a task switch. This suggests that although individuals who prefer parallel processing are able to process information in parallel throughout a task sequence, they clearly prefer to do so just before the task switch.

Multitasking, the simultaneous performance of multiple tasks, is a common behavior in both domestic and professional settings. In research, human multitasking is typically studied using rigorously controlled stimulus-response experiments (for reviews, see, e.g., Fischer & Janczyk, 2022; Janczyk & Kunde, 2020; Kiesel et al., 2010; 2022; Koch, Poljac, Müller, & Kiesel, 2018; Monsell, 2003). While these experiments are useful in identifying specific cognitive processes, they are less effective in exploring and explaining systematic variation across individuals. Nevertheless, the urge to understand individual differences in experimental paradigms has grown in recent years (e.g., Broeker et al., 2022; Miller & Schwarz, 2018; Rouder & Haaf, 2019). This interest has generated new paradigms and led to an accumulation of evidence supporting the existence of individual differences in multitasking (see, e.g., Brüning & Manzey, 2018; Damos, Smist, & Bittner 1983; Heidemann, Rickard, Schubert, & Strobach, 2020; Kubik, Del Missier, & Mäntylä, 2020; Laguë-Beauvais, Gagnon, Castonguay, & Bherer, 2013; Maquestiaux et al., 2008; 2018; Mendl & Dreisbach, 2022; Mittelstädt, Schaffernak, Miller, & Kiesel, 2021; Ruthruff, Van Selst, Johnston, & Remington, 2006; however, see Rouder, Kumar, & Haaf, 2023; Schuch, Philipp, Maulitz, & Koch, 2022 for recent critiques of the study of individual differences in cognitive psychology).

**Fig. 1:**
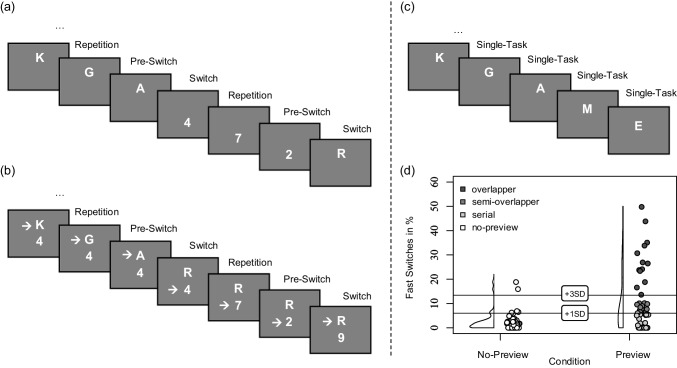
Exemplary sequences and conditions in task-switching paradigm and a schematic outline of the categorization of processing modes in task switching with preview. *Note*. The task sequence (**a**) shows a sequential task-switching paradigm with alternating runs. Participants classify letters (e.g., as vowels or consonants) and digits (e.g., as odd or even), switching tasks every three trials. Sequence (**b**) shows a task-switching sequence with preview (TSWP). The task sequence is identical to (a), but each trial additionally displays the upcoming task’s stimulus (preview), enabling participants to prepare for the next task in advance. Sequence (**c**) shows a condition in which participants solely respond to one task at a time (i.e., a single-task condition). The illustration (**d**) shows an exemplary distribution of individual fast switch rates (FSRs) across two experimental conditions: the classic sequential task-switching paradigm without preview (corresponding to the sequence shown in (a), labeled “No-Preview” in (d); n = 46), and the TSWP paradigm (corresponding to the sequence shown in (b), labeled “Preview” in (d); *n* = 45), both from Brüning and Manzey (2018). FSRs are displayed as percentages for consistency with previous work. They reflect the proportion of task switches executed faster than a predefined threshold (i.e., the 1^st^ RT quartile of single-task trials). Each dot represents one participant’s FSR, with density plots illustrating the distribution. Participants are further categorized based on their preferred processing mode: serial processors (*light gray circles*), semi-overlappers (medium gray circles), and overlappers (*dark gray circles*). *Horizontal lines* indicate +1 and +3 standard deviations from the mean FSR in the No-Preview condition, serving as reference markers

One particular paradigm that has recently been used to study individual differences in task processing when coordinating multiple tasks is the task switching with preview (TSWP) paradigm (Brüning & Manzey, 2018; Reissland & Manzey, 2016). In brief, the TSWP paradigm employs a fixed alternating task sequence (AAABBB) with the distinctive feature that the stimuli of both tasks are continuously visible (see Fig. 1B). Crucially, in this setting, only the stimulus of the currently cued Task A (B) is updated after each response, while the stimulus of the other Task B (A) remains visible as a preview for the next upcoming trial in Task B (A). Using this paradigm, evidence has accumulated that individuals differ in the extent to which they process the preview stimulus of the upcoming Task B (A) while still engaged in processing Task A (B) when switching between these tasks (e.g., Brüning & Manzey, 2018; Brüning et al., 2021; 2022; Reissland & Manzey, 2016). Some individuals do not utilize the preview of the upcoming Task B (A) for advance preparation and instead process the tasks serially (*serial processing mode*). Others seem to process the preview at least at the perceptual level^1^ in parallel with the ongoing processing of Task A (B) to prepare and optimize their mental shift towards Task B, resulting in shorter switch response times for Task B (*overlapping processing mode*).

The purpose of the present study is to improve our understanding of the time course and mechanisms of task switching preparation in individuals who use overlapping processing in task switching when a preview is provided. First, I review the basic mechanisms relevant to task switching. I then highlight key findings from TSWP experiments and discuss possible processing sequences underlying the preprocessing of upcoming tasks. Based on this, I present two experiments suggesting that overlapping preprocessing of stimulus information about an upcoming task in TSWP, when applied, occurs close in time to the actual task switch.

## Basic cognitive mechanisms involved in task-switching and task-switching with preview

In classical task-switching experiments, participants perform two tasks sequentially, typically involving rather arbitrary stimulus-response associations (e.g., categorizing digits as even or odd, classifying letters as vowels or consonants, or judging whether a number is greater or smaller than five). The main focus of these experiments is on the reconfiguration and interference processes that are enforced when switching between two tasks (e.g., Kiesel et al., 2010; Monsell, 2003). Various experimental paradigms have been developed to study task switching, such as alternating (e.g., Jersild, 1927; Rogers & Monsell, 1995), cued (e.g., Meiran, 2000), and voluntary task switching (e.g., Arrington & Logan, 2004). As the alternating paradigm is particularly relevant to this study, an illustrative trial sequence is shown in Fig. 1A. In so-called *mixed blocks*, participants encounter both tasks, for example, a letter and a number classification task. They switch between them after three responses to one task, resulting in a predictable pattern of *switch* trials (where the current task is different from the previous one), (pure) *repeat* trials, and *pre-switch* trials. In single-task blocks, participants perform only one task repeatedly (see Fig. 1C).

In the literature on task switching, switching costs are a key phenomenon. They are reflected in slower and often more error-prone responses during switch trials compared to repetition trials. Many theories have been developed to explain the precise mechanisms underlying switch costs (Allport, Styles, & Hsieh, 1994; Gilbert & Shallice, 2002; Logan & Bundesen, 2003; Mayr & Kliegl, 2003; Meiran, 2000; Rogers & Monsell, 1995; Rubinstein, Meyer, & Evans, 2001; Ruthruff, Remington, & Johnston, 2001; see Kiesel et al., 2010; Koch et al., 2018; Monsell, 2003, for reviews). What they all have in common is that they conceptualize tasks as mental representations, termed *task sets*, that encompass the respective endogenous or instructed goal, associated stimuli, and responses (see also, e.g., Koch et al., 2018). These task sets serve to guide task processing, as they specify how to select an appropriate response for the task goal (see also Koch & Kiesel, 2022). Importantly, individuals must update the task sets before they can fully process the new task following a task switch. The duration of this shift between task sets directly influences the efficiency of task processing during the switch trial, and thus the magnitude of switch costs.

One influential theoretical account postulates that switch costs can be attributed to active cognitive control processes involved in advanced task preparation (see, e.g., Logan & Bundesen, 2003; Mayr & Kliegl, 2000; 2003; Meiran, 2000; Rogers & Monsell, 1995; Rubinstein et al., 2001). Among the most prominent implementations of this view are those two-stage models that assume a time-consuming reconfiguration of task sets when switching between tasks (Rogers & Monsell, 1995; Rubinstein et al., 2001). This reflects an executive process, which involves the retrieval and reinstatement of the appropriate task set when switching to the respective other task. Research using the alternating-runs paradigm suggests that when participants can anticipate a task switch and are given sufficient time to prepare, they can reduce switch costs by engaging in preparatory task-set reconfiguration. According to Rogers and Monsell (1995), this reflects an endogenous component of task-set reconfiguration and constitutes the first stage. However, even with ample preparation time, switch costs are only partially reduced. A significant fraction typically remains – referred to as *residual costs*. Rogers and Monsell attributed these residual costs to an exogenous component of task-set reconfiguration that is triggered by stimulus onset, constituting the second stage. Alternatively, de Jong (2000) proposed the failure-to-engage hypothesis, suggesting that residual costs arise from a failure to fully engage in endogenous reconfiguration on some trials (see also Sohn & Anderson, 2001, for a similar implementation of an all-or-none process).

A second prominent account posits that switch costs result from aftereffects of the previous task set, or “task-set inertia”—a concept referring to passive interference due to task-set decay (Allport et al., 1994; see also Wylie & Allport, 2000; Grange, 2016, for alternative explanations such as learned stimulus associations or temporal task-set distinctiveness). This view holds that switch costs occur because the cognitive system requires additional time to “settle into the correct response representation” (Gilbert, 2005, p. 95), with increased competition between task sets. This competition is not immediately resolved, influencing subsequent repetition trials as well. In sum, both accounts are supported by evidence (e.g., findings from the task-cueing paradigm, which show reconfiguration costs independent of decay, Koch, 2001; Meiran, 1996; Meiran, Chorev, & Sapir, 2000). Notably, these theoretical accounts in task-switching research specifically address switch costs in serial task processing, where experimental procedures are designed to enforce serial processing and exclude parallel task processing.

A relatively novel approach to task switching research, which allows the reduction of switching costs through parallel processing, is the TSWP paradigm (Reissland & Manzey, 2016; Brüning & Manzey, 2018; Brüning et al., 2021, 2022; Schindler et al., 2023). As outlined above, its general procedure capitalizes on the alternating-runs design, but with the extension of a preview of the next task-switch stimulus (see Fig. 1B). Specifically, participants are always presented with two vertically separated stimuli, one for each task. Which of the two tasks is currently relevant is indicated by an arrow pointing to one of the task stimuli, thus guiding participants through the task-switching procedure. After a response is provided to the currently relevant task, only that stimulus is updated, while the other stimulus remains. After three responses to the currently relevant task, the arrow always moves to the other task stimulus, indicating that this is now the relevant task. Thus, like the classical alternating runs paradigm, this experimental procedure allows participants to anticipate when to switch, but additionally gives them a preview of the specific task stimulus to which they must respond after the switch. However, participants are not explicitly instructed to use or ignore the preview. Instead, they are instructed to optimize their overall performance, often coupled with a monetary incentive. How they achieve optimal performance remains up to them.

Using the TSWP paradigm, evidence has accumulated that individuals differ in whether they tend to process tasks serially, that is, as if no preview were available, or whether they take advantage of the preview in order to optimize switch times (Brüning & Manzey, 2018; Brüning et al., 2021; Reissland & Manzey, 2016). The former participants usually produce average switch costs in the range known from the classical alternating-runs paradigm without preview. In contrast, the latter group produces a significant proportion of extremely fast switches compared to all switches (i.e., a high fast switch rate, FSR), thereby considerably reducing their switch costs. This suggests that they have fully or at least partially processed the previewed stimulus while working on the still-relevant task, which enabled them to respond very quickly to this stimulus after the switch (“fast switch criterion” to separate the used processing mode^2^; Brüning & Manzey, 2018; Brüning et al., 2021). For a typical distribution of FSRs see Fig. 1D.

Accordingly, these individuals may be able to keep both tasks sets active at the same time, at least to some extent, by processing and storing information about the upcoming task in their working memory while they are still engaged in processing the current task. The study by Brüning and Manzey (2018) has also suggested that individuals who engage in overlapping processing of the preview tend to have higher working memory capacity than those who adhere to strictly serial task processing. Further experiments suggest that individuals who engage in overlapping processing may also be less susceptible to between-task interference (Brüning et al., 2022). Furthermore, when given freedom of task choice, participants exhibiting a higher FSR in the TSWP paradigm, also exhibit a higher switch rate in a voluntary task-switching paradigm (i.e., they switch tasks more frequently) than participants showing a low FSR in the TSWP paradigm (Brüning et al., 2021). That is, the differences in processing modes translate into free-choice behavior. These behavioral patterns are highly stable and persist even under increased between-task interference (Reinert & Brüning, 2022). Under favorable conditions, however, a high FSR even results in a net performance gain in mixed versus single-task blocks (Brüning, Mückstein, & Manzey, 2020).

### Time course of overlapped processing and the present study

Although individual differences in the fast switch metric have been demonstrated time and again (e.g., Brüning & Manzey, 2018; Brüning et al., 2021; 2022; Reissland & Manzey, 2016), the time course of overlapped processing is still unclear. The TSWP paradigm’s less controlled procedure offers greater freedom for overlapping processing of the tasks. However, this flexibility also presents the challenge of determining exactly when individuals who tend to preprocess an upcoming task begin to use the available preview (i.e., are *overlapping processors*, or *overlappers* for short). This also reflects a more general question: how much preparation time is necessary for effective parallel processing at a task level (see also Brüning et al., 2022), and how early humans begin to exploit available information to prepare for an upcoming task switch. The present study reports two experiments aimed at disentangling two conceivable time courses of task preparation that relate to two aspects: preview length and preview position. In Experiment 1, participants performed a version of the TSWP paradigm with varying preview length (i.e., for one to three trials). More precisely, in the original version of the TSWP paradigm, participants could use the preview information by selecting any appropriate time while working on the currently relevant task. That is, the preprocessing of the preview stimulus could, for example, occur in parallel with the encoding of the currently relevant stimulus or the execution of the corresponding response on any of the three trials. In this case, the time of preview usage could vary across the AAA/BBB sequence, and the preview should be easier to use the longer the stimulus is available – that is, the wider the time window for selecting an appropriate moment for overlapping processing. As a consequence, one could expect that FSRs increase with longer time windows. However, while it is important to first examine effects of preview duration – since the available time fundamentally constrains opportunities for overlapping processing and thus provides a necessary foundation for understanding preview usage – manipulating duration inevitably also affects preview position. Specifically, shortening the preview necessarily changes its onset within the task sequence, making preview length and preview position inherently confounded. Therefore, in Experiment 2, participants received the preview at discrete locations in the task-switching sequence to test whether preview usage might be specifically localized to a single position during the sequence. In this case, individuals might only process the preview stimulus in parallel with stimulus encoding or motor execution in a specific trial of the currently relevant task. For example, they could already process the preview while switching to the new task (i.e., overlapping with the switch trial), or they could process the preview in the last trial of a sequence (i.e., the pre-switch trial) to reduce working memory demands. Accordingly, overlapping processing may be particularly effective when the preview is used at a specific moment in the task sequence—such as shortly after responding to the final Task A stimulus in an AAABBB sequence, when attention has not yet fully disengaged from Task A and the participant can begin processing the upcoming Task A preview in parallel with initiating Task B. Note that during the planning of the study, no performance differences were expected for those individuals who stay with a strictly serial task processing (i.e., *serial processors*, or *serials* for short), based on the assumption that they do not benefit from the preview. As it is difficult to make assumptions about the preview’s systematic effect for participants who only occasionally engage in its use for overlapping processing (i.e., *semi-overlapping processors*, or *semi-overlappers* for short), such expectations are expressed with caution in the following.

## Experiment 1

**Fig. 2:**
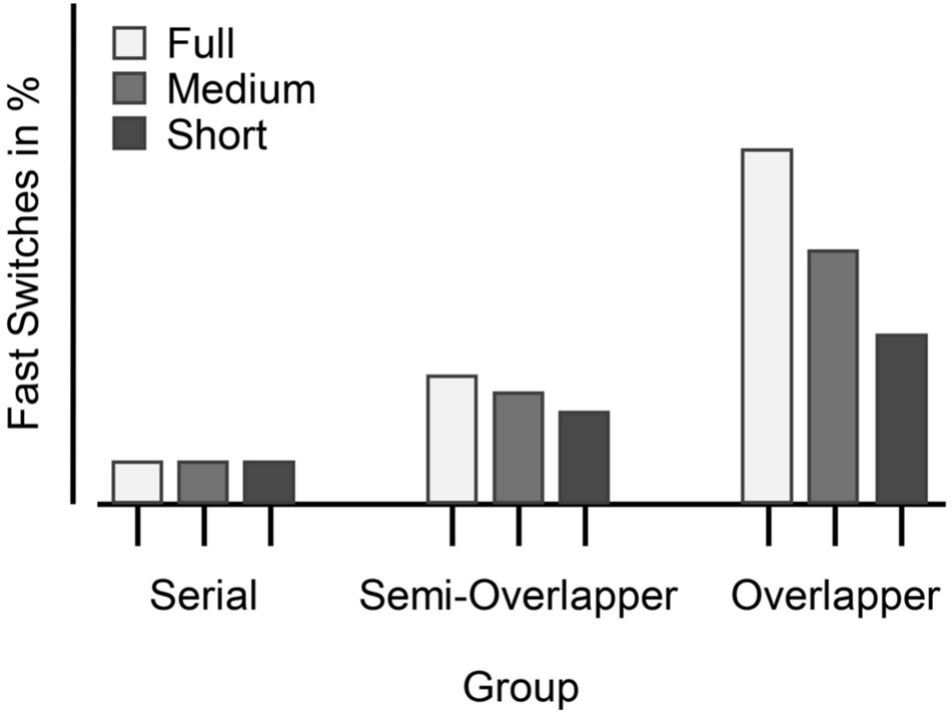
Expected mean fast switches (in %) for Experiment 1. *Note*. Depicted are the expected average percentage of fast switches as a function of processor group (Serial, Semi-Overlapper, Overlapper) and preview-length condition (Full, Medium, Short, see TSWP versions and procedure for details). While fast switch rates are expected to remain low and stable across preview conditions for serial processors, a decreasing trend is expected for overlappers as preview length shortens. Semi-overlappers are expected to fall between these two groups, showing moderately fast switch rates across conditions

In Experiment 1, the length of the preview was varied blockwise by starting the preview either (i) with the first task stimulus in a sequence (i.e., with the switch trial, “full-preview condition”; equal to the original version of the paradigm), (ii) concurrent with the second task stimulus (i.e., the repetition trial, “medium-preview condition”), or (iii) with the third task stimulus (i.e., the pre-switch trial, “short-preview condition” ). In all cases, the preview stimulus remained visible until it finally became the relevant task stimulus after the next task switch. The expected mean FSRs per processing mode in each condition are illustrated in Fig. 2. As serial processors, by definition, make no use of the preview, I predicted that their FSRs remain low, regardless of when the preview is presented. For participants who consistently use the preview for overlapping processing, the FSR should depend on when they process the preview. Because longer presentation times of the preview provide more degrees of freedom for these participants to choose an appropriate time for parallel processing of the preview stimulus, the FSR is expected to depend directly on how long the preview is available. This would be reflected in the following pattern: FSR(i)> FSR(ii)> FSR(iii). Because semi-overlappers only occasionally engage in overlapping processing, it is difficult to derive a clear hypothesis for this group. However, to the extent that they do engage in overlapping processing, any descriptive differences in performance that may emerge should be similar in direction to those observed for consistent overlappers.

### Method

#### Transparency and openness

I report how I determined the sample size, all data exclusions, all manipulations, and all measures in the study, and I follow JARS (Kazak, 2018). All raw data are available at https://osf.io/ry3pz/?view_only=aa21a94eb9e64c8a85c1d735b225148a. Data were primarily analyzed using R (R Core Team, 2022), most recently with version 4.3.1, and with the packages tidyverse (Wickham et al., 2019, v. 2.0.0), schoRsch (Pfister & Janczyk, 2022, v. 1.10), ez (Lawrence, 2016, v. 4.4-0), Rmisc (Hope, 2022, v. 1.5.1), WRS2 (Mair & Wilcox, 2020, v. 1.1-6). This study was not preregistered.

#### Participants

Forty-eight volunteers participated in this experiment (22 female, 25 male, and one non-binary individual; between 18 and 35 years, mean age = 26^3^, 42 right-handed, six left-handed). Participants reported normal or corrected-to-normal vision, were naïve regarding the hypotheses of this experiment, and signed informed consent before data collection. Participants received either course credit or 12€  for participation, plus a performance-based bonus of up to  €5 for correct responses in the TSWP. As the tasks in this paradigm are performed in fixed time blocks (e.g., 60 s), participants can perform more correct trials by responding faster, resulting in a higher bonus.

The sample size was based on a power analysis using G*Power 3.1 (Faul, Erdfelder, Buchner, & Lang, 2009) for the effect of interest, that is, the interaction between preview length (repeated measure) and processing group (between-participants factor). Since there are yet no empirical data regarding an effect of varying length of preview display for TSWP groups on which I could base the estimation of a reasonable effect size, I assumed effects of $\eta _p^2 =.06$ to be of relevance (i.e., a ‘medium’ effect size according to Cohen, 1988; although which effect sizes are of relevance may vary across research contexts, Fritz, Morris, & Richler, 2012). Assuming a power of $1-\beta =.90$ and $\alpha =.05$, the overall sample size required to detect such an effect was $n = 45$. Note that this power analysis represents an idealized and approximate approach, based on the assumption of equal group sizes and the use of a standard ANOVA framework. However, given that preferred processing modes vary naturally across individuals, some degree of group size imbalance is likely and difficult to anticipate precisely during the planning stage. To address the imbalance in group sizes, I used robust ANOVA methods that account for potential heteroscedasticity (see the Data analysis section for details). Further, I increased the sample size to 48 to account for dropout and to achieve counterbalancing of conditions. However, no complete datasets had to be excluded as all participants met the criteria of having less than 20% errors in any block of any condition and producing a reasonable number of trials (i.e., at least 40 trials per task and condition). The final sample size remains within the range of previous studies using the TSWP to assess individual preferences (Brüning & Manzey, 2018; Brüning et al., 2021).

#### Tasks

A scheme of the stimulus presentation in the TSWP paradigm is depicted in Fig. 1 (sequence B). Participants performed a digit and a letter classification task that are commonly used in the task-switching literature (e.g., Rogers & Monsell, 1995). In the digit task, a single digit was called for classification according to its parity (2, 4, 6, 8 vs. 3, 5, 7, 9). In the letter task, participants had to categorize a single capital letter as a vowel or consonant (A, E, I, U vs. G, K, M, R). Within each task-switching block, the tasks were presented in a predictable AAABBB pattern, resembling an alternating runs scheme (Rogers & Monsell, 1995). With three repetitions for each task, trials in task-switching blocks can be distinguished into switch (A**B**BBA), pure-repetition (AB**B**BA), and pre-switch (ABB**B**A) trials.

#### Apparatus and stimuli

Stimulus presentation and response collection were controlled by standard PCs. The stimuli were displayed in white (RGB = 245, 245, 245; font size = 24 px) on a dark gray background (RGB = 90, 90, 90) on an Acer LCD monitor (1280 $\times$ 1024 px, 60 Hz refresh rate). Within single-task blocks, only one stimulus was presented in the center of the screen. In task-switching blocks, stimuli for both tasks were presented simultaneously and in close spatial proximity, one just above and one just below the center of the screen. In the trials where no preview was displayed, a \$  sign was presented instead of the respective stimulus to keep the visual features of the paradigm constant and to avoid saliency effects. A white arrow pointing at either the digit or the letter indicated the current task. Importantly, the digits and letters were always visible, and a response in one task did not change the stimulus in the other task. Responses were collected using a standard USB keyboard with each hand assigned to one task. The keys ‘K’ and ‘L’ were used with the index and middle finger of the right hand, respectively. The keys ‘S’ and ‘A’ were used with the index and middle finger of the left hand, respectively. Task-hand assignment was counterbalanced across participants. Additionally, relevant keys were marked by colored points for easier recognition. In single-task and in task-switching blocks, the stimuli remained on the screen until a response for the corresponding task was registered. The stimulus was then immediately replaced by another randomly drawn stimulus of the corresponding task. Note that the response-stimulus interval was zero, and immediate stimulus repetition was not possible.

#### TSWP versions and procedure

The experiment was conducted in a laboratory of the Technische Universität Berlin. A maximum of three participants were tested simultaneously at individual PC workstations. These were separated by opaque screens, and participants were provided with earplugs to minimize distractions. A session started with general instructions and signing the consent form. Instructions were presented in written form on the computer screen, were read self-paced, and emphasized speed while maintaining errors at a low rate. All tasks were performed in blocks for a fixed time (e.g., 60 s), that is, tasks could not be completed in a shorter time frame.^4^

**Fig. 3:**
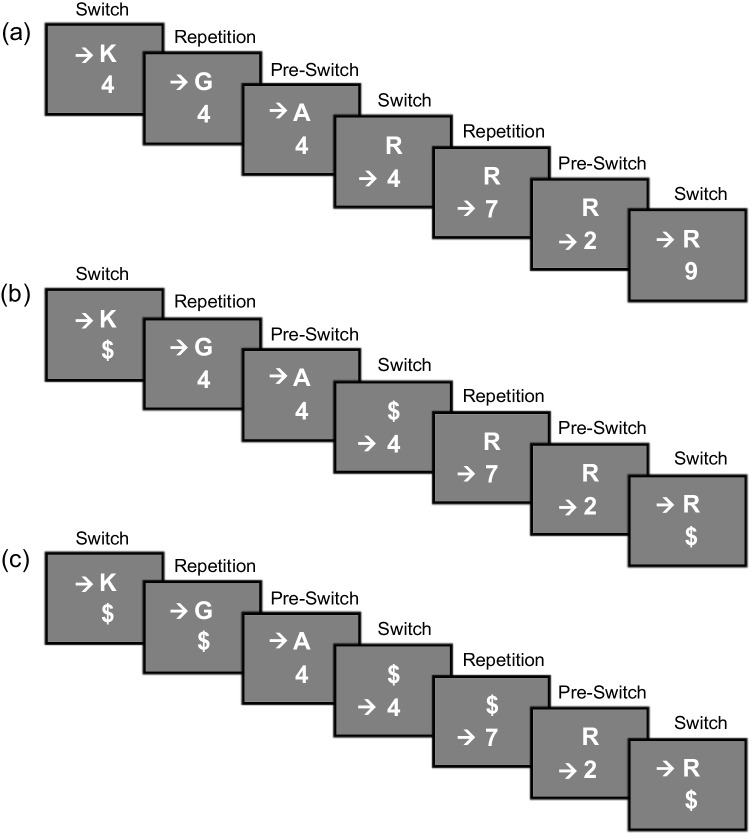
Task switching with preview conditions with different preview lengths. *Note*. Sequence (**a**) shows the standard task switching with preview paradigm, which serves as the *full-preview* condition in Experiment 1. Sequence (**b**) shows the *medium-preview* condition, in which participants are presented with a shorter preview starting from trial 2. Sequence (**c**) shows a condition in which participants are presented with the preview only in the last trial of a sequence, that is, the *short-preview* condition

Participants performed the TSWP paradigm in three different versions (see Fig.  3), which varied between blocks. A Williams design (balanced Latin square) was used to counterbalance the order of conditions across participants. This design ensures that each condition appears equally often in each serial position and is preceded and followed equally often by every other condition, thus controlling for both position and immediate carryover effects. Participants performed a TSWP version in which the preview presentation started concurrently with the first task stimulus of an **A**AA/**B**BB sequence. That is, the preview stimulus occurred immediately after the pre-switch trial response with the onset of the next switch trial. In this case, the preview is visible in all three trials (i.e., *full-preview* condition, see Fig. 3A). In addition, participants performed a second TSWP version in which the preview started concurrently with the second task stimulus of an A**A**A/B**B**B sequence. That is, the preview stimulus was not yet displayed in the switch trial, but the presentation started in the pure-repetition trial. Accordingly, the preview is only visible in the second and third trial of the alternation sequence (i.e., *medium-preview* condition, see Fig. 3B). In the third TSWP version, the preview presentation started concurrently with the third task stimulus of an AA**A**/BB**B** sequence. Participants received the preview only in the pre-switch trial. That is, the preview was only available for one trial before the task switch (i.e., *short-preview* condition, see Fig. 3C). In all cases, the preview stimulus remained visible until it finally became the relevant task stimulus after the next task switch.

The experiment consisted of a practice and an experimental phase. The practice phase included single-task and task-switching blocks to familiarize participants with each condition. Participants always started with the performance of each classification task in isolation (each 30-s familiarization block, 60-s practice block). They were then acquainted with each preview-length condition in a 30-s familiarization block, followed by a 120-s practice block. While participants always started with the single-task blocks and then continued with the task-switching blocks, the order of the single-task blocks and the order of the preview-length conditions were counterbalanced across participants.

In the subsequent experimental phase, participants completed three experimental runs, one for each preview-length condition. Each run started with a 60-s task-switching block to prepare participants for the upcoming preview-length condition. After a brief 20-s break, participants performed the respective preview-length condition for two blocks of 120 s, followed by one 60-s block of each single-task. Note that the order of preview-length conditions in both the practice and experimental phases for each participant followed the assignment according to the respective version of the Williams design. The order of single tasks was counterbalanced across runs. Single-task blocks were included to control for practice effects, as the identification of “fast switches” as the critical variable for identifying overlapping processing requires a stable reference in terms of single-task response speed (see Data analysis). After each block, participants received feedback about the number of their responses as well as the number and percentage of correct responses. In total, the experiment lasted about 75 min.

#### Data analysis

Data were analyzed mirroring previous studies (e.g., Brüning et al., 2021). Accordingly, data were pre-processed in the following steps: As trials of the TSWP paradigm do not have a time limit, trials with response times (RTs) longer than 5000 ms were excluded first. For the analysis of RTs, only correct trials were considered. Subsequently, trials with RTs deviating more than 2 standard deviations (*SDs*) from the participant’s mean (*M*) correct RT, computed separately for each combination of trial type and experimental block, were excluded. In total, 4.75% ($SD=0.89\%$) trials were discarded in the *full-preview* condition, 4.42% ($SD=0.79\%$) trials in the *medium-preview* condition, and 4.70% ($SD=0.96\%$) trials in the *short-preview* condition. On average, participants performed 172 trials in single-task blocks and 317 trials in task-switching blocks in the full-preview condition, 174 trials in single-task blocks and 322 trials in task-switching blocks in the medium-preview condition, and 170 trials in single-task blocks and 319 trials in task-switching blocks in the short-preview condition (see Appendix A for more details).

To assess how often individuals use the preview for overlapping processing, the proportion of so-called fast switches was determined for each participant in each condition (see also Brüning & Manzey, 2018, p. 98; Brüning et al., 2021, p. 583). Fast switches are defined as correct switch trials that meet two conditions. First, the RT is at least as fast as the 25% fastest single-task responses in the upcoming single-task block. Note that single-task blocks are repeated after each task-switching block, and thus the comparison between switch RTs and single-task RTs remains comparable across experimental runs (i.e., remains stable regardless of potential practice effects). Second, fast switches are not the result of compensatory prolongation in the three trials preceding a task switch. To quantify this, intervals consisting of three consecutive trials of the same task (e.g., “AAA” or “BBB”) immediately before a task switch were identified within the alternating task sequence (“AAABBBAAA”). For each interval, the total time taken to respond to all three trials was calculated. All intervals preceding non-fast switch trials were then averaged (i.e., switches slower than 25% of the fastest single-task responses in the subsequent single-task block). The key idea is that responses preceding a fast switch may, on average, contain additional time used to process the preview (i.e., in terms of compensatory prolongation), whereas responses preceding non-fast switches should not. By contrasting the interval preceding each fast switch with the average interval preceding all non-fast switches, a potential compensatory delay can be quantified. If the compensation outweighs the performance benefit on a switch trial (i.e., is greater than the difference between the switch RT and the average single-task RT), then the switch is no longer considered a fast switch. I then computed the FSR, defined as the proportion of correct fast switches relative to all correct switch trials. This metric was used to classify the participants according to their preferred mode of processing. If a participant is a *(semi-)overlapping processor*, its FSR is expected to be relatively high. If a participant is a *serial processor*, its FSR is expected to be relatively low. To determine which FSR could be considered “high” or “low,” I took the distribution of FSR under a task switch condition without preview, which was provided in the Brüning and Manzey (2018) dataset. Based on the mean FSR in the *full-preview* condition (i.e., the standard condition in previous studies), participants were then classified as overlapping, semi-overlapping, and serial processors, respectively (see Fig. 1D, and for more detailed information on the derived cutoffs for the classification as well as an illustration depicting the described comparisons see the Appendix in Brüning et al., 2022).

In order to take the individual differences in the preference for processing modes into account, the classification of participants in the (standard) *full-preview* condition served as a between-participants variable (Brüning & Manzey, 2018; Brüning et al., 2021, 2022). Mean FSR of all three preview-length conditions was submitted to an analysis of variance (ANOVA) with preview-length (full vs. medium vs. short) as a repeated measure and processing mode (overlappers vs. semi-overlappers vs. serials) as a between-participants variable. Note that, as the categorization is a basis for the analysis, the main effect of categorization on the FSR is not of interest but will only be reported for the sake of completeness. In case the sphericity assumption was likely violated, *p* values were corrected according to the procedure by Greenhouse and Geisser (1959), and the corresponding $\epsilon$ is reported.

As group sizes could vary due to naturally occurring differences in processing preferences across individuals, this could affect analyses involving the between-participants factor processing mode. To account for this, and for potential violations of normality and homoscedasticity, robust methods based on trimmed means ($M_t$) were used (Mair and Wilcox, 2020). Specifically, robust ANOVAs on FSRs were performed using the WRS2::bwtrim function (20% trimmed means) from the WRS2 package in R (see the Transparency and openness section). Note that in robust mixed ANOVA methods involving only two factors, test statistics can be approximated using an adjusted F-distribution, and corresponding degrees of freedom $(df_{1}, df_{2})$ can be calculated based on the trimmed-means variance estimates. In contrast, robust ANOVAs with more than two factors employ a Q-statistic, which is evaluated against simulation-derived critical values; therefore, degrees of freedom are not defined and not reported. Effect sizes were computed via the bw.es.A, bw.es.B, and bw.es.I functions, which return the robust effect size estimates Kulinskaya–Morgenthaler–Staudte (*KMS*) for between-participants factors and Algina–Keselman–Penfield (*AKP*) for within-participants factors and interactions. These metrics can be understood as a robust heterocedastic analog of Cohen’s *d* with values of 0.20, 0.50, and 0.80 corresponding to small, medium, and large effect sizes (see Wilcox, 2023, for a guide to robust statistical methods). These robust methods offer more reliable results in the presence of unequal group sizes and violations of standard ANOVA assumptions.

### Results

As is typical for simple RT tasks in the TSWP paradigm, the mean percentage errors (PEs) were very low. This was the case for the PEs in the single-task blocks in the *full-preview* condition, $M_{fullP} = 3.9$ and $SD_{fullP} = 3.1$, as for the *medium-preview* condition, $M_{medP} = 3.9$ and $SD_{medP} = 3.2$, and for the *short-preview* condition, $M_{shortP} = 3.5$ and $SD_{shortP} = 3.1$. The PEs in the task-switching blocks were even slightly lower in the *full-preview* condition, $M_{fullP} = 2.9$ and $SD_{fullP} = 2.5$, as for the *medium-preview* condition, $M_{medP} = 2.5$ and $SD_{medP} = 2.0$, and for the *short-preview* condition, $M_{shortP} = 3.0$ and $SD_{shortP} = 2.5$. Although overall PEs were relatively low, additional analyses were conducted to examine potential speed–accuracy trade-offs for each experimental condition and processing mode (see Appendix B for details). These yielded a significant main effect of trial type, with only small absolute differences in PEs of about $0.8\%$ to $0.9\%$, but no main effects of condition or processing mode. There were also no interaction effects. Thus, the small differences in PEs are unlikely to fully account for the substantial differences reported in the following for RTs and FSRs.

**Fig. 4:**
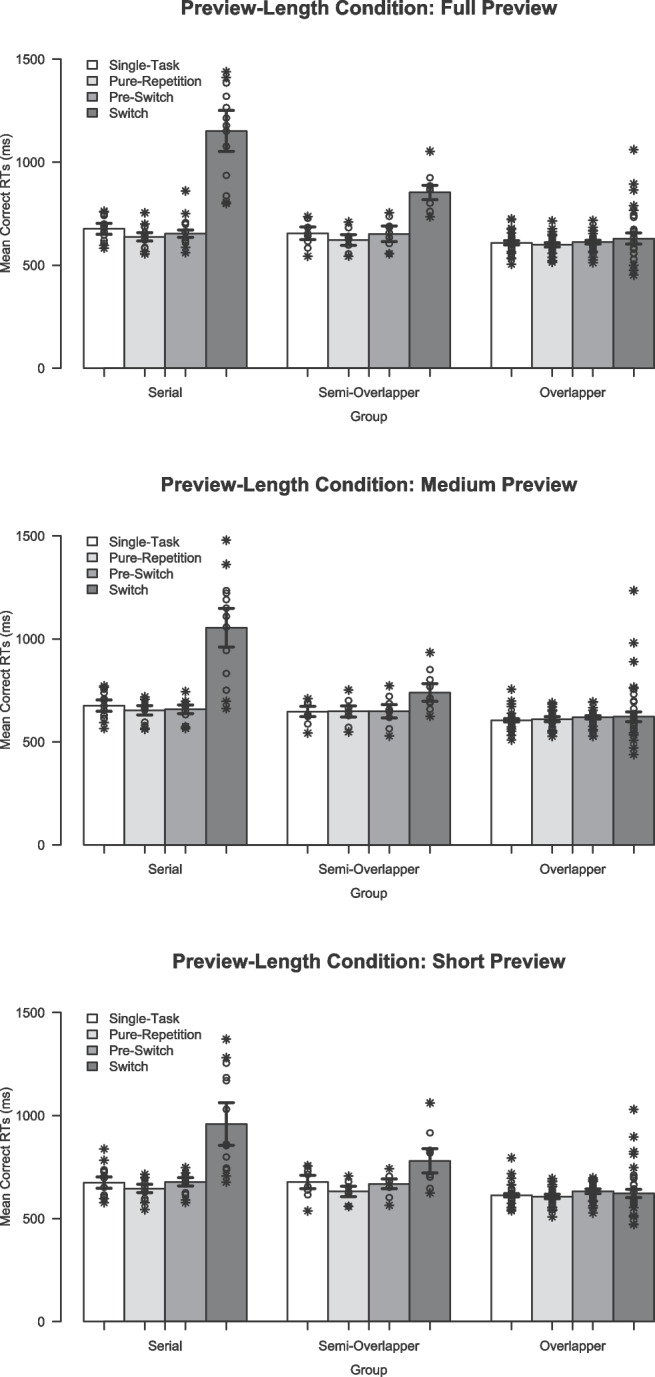
Mean response times ($M_t$) per trial type for each processing mode in each preview-length condition. *Note*. Depicted are the trimmed mean response times (RTs, in milliseconds) as a function of processor groups and preview-length condition. *Error bars* indicate ± one standard error calculated separately for each Trial Type in each Group $\times$ Preview-Length Condition. *Circles*/*crosses* represent individual data included/excluded in the calculation of trimmed means (20% criterion)

#### Processing modes and RTs

Based on the distribution of FSRs in the *full-preview* condition (i.e., the standard condition in the TSWP paradigm), 26 participants were classified as overlappers, 9 as semi-overlappers, and 13 as serials. Based on previous research, it was expected that the number of participants in each subgroup of processing modes would differ due to natural variations in preferences for the different processing modes. However, a notable difference in the subgroups’ size can pose an impediment to the statistical analyses. To counteract this issue, I used robust implementations of heteroscedastic ANOVAs based on trimmed means ($M_t$) for all statistical comparisons involving the between-group factor (i.e., the R package WRS2), as suggested by Mair and Wilcox (2020). Note that I will therefore report the $M_t$ in all such cases and the arithmetic mean ($M_a$) otherwise, which will be noted.

Trimmed means and individual RTs per trial type for each processing mode in each preview-length condition are visualized in Fig. 4. In the *full-preview* condition, RTs were generally quite short (i.e., 600 ms to 677 ms) in the single-task, pure-repetition, and pre-switch trials. However, switch RTs showed considerable variance across processor subgroups. As is typical for the TSWP paradigm with full preview, *serial processors* exhibited pronounced switch costs, with RTs prolonged by approximately $\hat{\Delta } = 495$ ms, whereas *overlapping processors* showed only a minimal increase of around $\hat{\Delta } = 23$ ms compared to the other trial types. The switch RTs produced by *semi-overlapping processors* were in between these extremes, that is, they were only moderately prolonged by $\hat{\Delta } = 210$ ms. Overall, this resulted in an expectable interaction between processing mode $\times$ trial type, $F(6,28) = 6.15$, $p <.001$, $AKP = 0.99$.

When comparing RTs produced in the *full-preview* condition with RTs in the *medium-preview* condition and the *short-preview* condition, the RTs were similarly short in the single-task, pure-repetition, and pre-switch trials. For the switch RTs a divergent pattern emerged, which is also reflected in an interaction between preview-length condition and trial type, $$F(6, 270) = 13.73 $$, $$p <.001$$, $$\eta _p^2 =.23$$. The differences in trial types across preview-length conditions were not consistent among processor subgroups. Remarkably, the overlappers showed similar RT patterns across trial types in the *medium-preview* and *short-preview* conditions compared with the *full-preview* condition, that is, they showed no substantial prolongations in switch RTs in any of the different preview length conditions ($$\hat{\Delta } = 2$$ ms to $$\hat{\Delta } = 17$$ ms). Although serial and semi-overlapping processors exhibited longer RTs on switch trials compared to all other trials in the *medium-preview* and *short-preview* conditions, their switch RTs were surprisingly slightly shorter in these blocks of shorter preview presentations than in the *full-preview* condition.

#### Fast switch rates

**Fig. 5:**
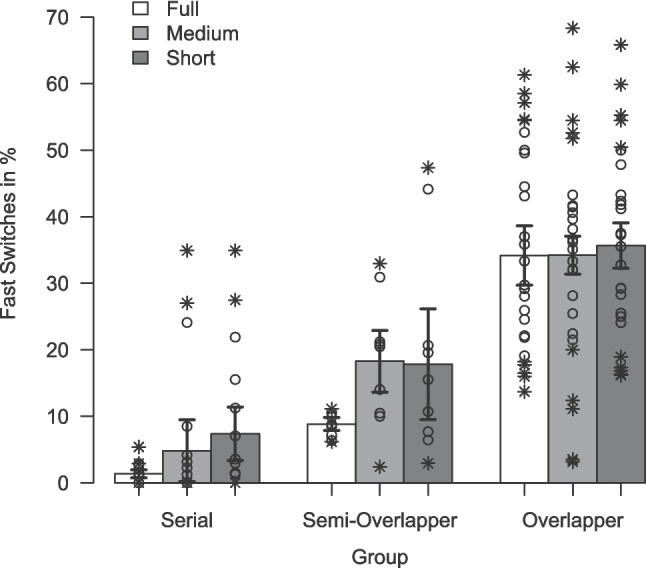
Mean fast switches (in %) for Experiment 1. *Note*. Depicted are the average percentage fast switches as a function of processor groups and preview-length condition (calculated on trimmed means). *Error bars* indicate ± one standard error calculated on trimmed means, separately for each Group $\times$ Preview-Length Condition. *Circles*/*crosses* represent individual data included/excluded in the calculation of trimmed means (20% criterion)

While the summary of mean RTs provides an overview of the processor groups’ general response speed across trial types and conditions, the FSR for each condition offers a more detailed measure of participants’ peak performance by indicating the proportion of switch responses that exceed a specific threshold (i.e., at least as fast as the 25% fastest single-task responses). Remember that an individual’s FSR reflects the percentage of switches to which the individual responded so quickly that it can be assumed that the switch stimulus was preprocessed prior to the switch in preparation for that response (i.e., there was some overlapping processing of both tasks). The mean FSR ($M_t$) per processing mode in each condition is visualized in Fig. 5. Not surprisingly, as it is based on the categorization procedure, the corresponding robust ANOVA indicated a main effect of processor group with generally higher FSRs for overlappers ($34.7\%$) than for semi-overlappers($12.7\%$), and serial processors ($2.7\%$), $F(2, 29.53) = 26.98$, $p <.001$, $KMS = 0.80$. Neither the main effect of preview-length condition ($p =.100$) nor the interaction of processor group and condition ($p =.253$) was significant in the robust ANOVA. Descriptively, the FSRs mirrored the result pattern observed in switch RTs across preview-length conditions. For overlapper, FSRs were similarly high in all three preview-length conditions (full: $34.2\%$, medium: $34.2\%$, short: $35.7\%$). For serial and semi-overlapper, FSRs were lower in the *full-preview* condition (serial: $1.4\%$, semi-overlapper: $8.8\%$) than in the *medium-preview* condition (serial: $4.8\%$, semi-overlapper: $18.3\%$), and the *short-preview* condition (serial: $7.4\%$, semi-overlapper: $17.8\%$).

#### Fast switch rates: exploratory analysis

While the FSRs for overlapping processors are clearly on a similar level in all three preview-length conditions, the FSRs of the semi-overlapping and serial processor groups appeared to vary descriptively between conditions. For the serial processor group, no differences were expected, as outlined in the introduction, based on the assumption that they do not benefit from the preview. For the semi-overlappers, no explicit hypothesis was stated, as their occasional engagement in overlapping processing made it unclear whether a systematic effect of preview length would emerge. However, given that they do show at least some incidences of overlapping processing, a similar – albeit less pronounced – pattern as observed in overlapping processors (i.e., increasing FSRs with longer preview lengths) would have been plausible. Contrary to this expectation, the observed pattern pointed in the opposite direction, with higher FSRs occurring at shorter preview lengths. To determine whether these unexpected differences were statistically meaningful or merely due to noise, I conducted two exploratory one-way ANOVAs, one for each processor group. These analyses revealed significant effects of preview length in both groups (semi-overlapper: $F(2, 16) = 4.02$, $p =.038$, $\eta _p^2 =.33$; serial: $F(2, 24) = 5.11$, $p =.030$, $\eta _p^2 =.30$).

### Discussion

Experiment 1 was designed to test to what extent the length of the preview in the TSWP paradigm is critical for its utilization and thus for the occurrence of overlapping processing. For this purpose, a *medium-preview* and a *short-preview* condition have been introduced in addition to the *full-preview* condition used before (Brüning & Manzey, 2018; Brüning et al., 2021, 2022; Reissland & Manzey, 2016). While the preview is always available in the *full-preview* condition, it is only displayed in the second and third trials of a sequence in the *medium-preview* condition, and only in the third trial immediately preceding the switch trial in the *short-preview* condition.

For the *full-preview* condition, the FSR pattern and the resulting numbers of serials, semi-overlappers, and overlappers obtained is highly similar to previous results (Brüning & Manzey, 2018; Brüning et al., 2021, 2022; Reissland & Manzey, 2016). The resulting main effect of the group is therefore more of a sanity check. However, the fact that there was neither an effect of nor an interaction effect with the preview length condition was surprising. Descriptively, there are also no visible differences in the FSRs between the conditions for overlappers. This suggests that the length of the preview is not the decisive feature. Instead, overlapping processing is possible even with a medium to short preview and thus a rather short preparation phase.

**Fig. 6:**
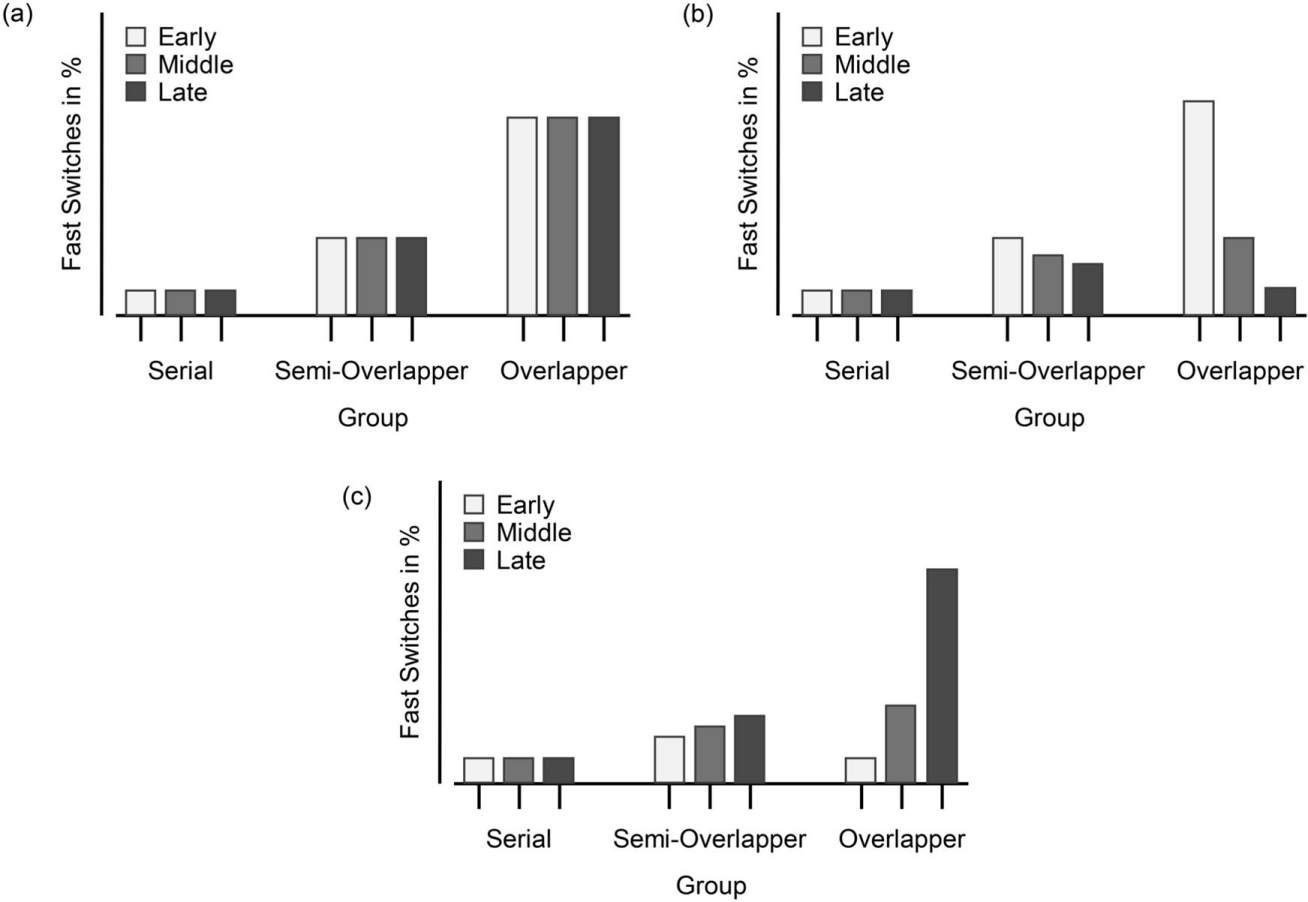
Expected mean fast switches (in %) for Experiment 2. *Note.* Depicted are the expected average percentage of fast switches as a function of processor group (Serial, Semi-Overlapper, Overlapper) and preview-position condition (Early, Middle, Late). Each panel plots the expected average percentage of fast switches based on a specific hypothesis about when the preview is processed within the sequence: (**a**) the preview is used at any of the three positions, (**b**) it is processed as soon as it is presented (i.e., preferably on the first trial or, at the latest, on the second), or (**c**) it is processed in parallel toward the end of the sequence (i.e., on the second trial or, preferably, the last). For serial processors, fast switch rates are expected to remain low across all three conditions. Semi-overlappers are anticipated to show intermediate patterns

Critically, though, the brief presentation of the preview always occurred only on the last trial before the switch, leading to a confound between preview length and position. Accordingly, there are two possible ways in which the preview could have been used. First, overlappers may be capable of using the preview very rapidly and independently of the exact position. This would be plausible because overlappers achieved fast switches irrespective of the length of the preview, even if it only occurred during a single trial. However, all preview length conditions involved the preview being present on the third trial of a sequence (i.e., applying to the *short-preview*, *medium-preview*, and *full-preview* conditions). Therefore, a second possibility is that the preview was used only just before the switch. The results so far do not allow us to determine whether the results of Experiment 1 are due to overlappers being able to rapidly use the preview at any time, or whether they specifically used the preview during the last trial before a switch.

In addition to the consistently high FSRs for overlappers, I did not expect to see an increase in FSRs for serials and semi-overlappers with shorter previews. This increase, which is quite apparent in the descriptive results, was also reflected in statistically significant differences in an exploratory analysis. A higher number of incidences of preview use when actually less preview is provided might imply that for serial processors, displaying the preview early in the sequence may be more distracting than helpful. That is, the preview shown early in the sequence might rather interfere with their processing of the currently relevant task. Therefore, replacing the preview with a \$ character early in the sequences in Experiment 1 may have reduced this distraction and potentially related interference because it did not require a response. However, whether this indeed results in an active use of preview or reflects more of a passive processing due to lowered inhibition of the second task, remains elusive for now. Since the results and interpretation are purely exploratory, it can only be tentatively speculated at this point, and a replication of this pattern is needed first.

Before drawing further conclusions, I present an additional experiment that further disentangles the aspects of preview length and position for overlappers and provides a replication of the increase in FSR for serial and semi-overlapping processors. Specifically, in the second experiment, the preview was not presented for varying durations, but only simultaneously with the first, second, or third stimulus of the respective task. Once the participant responded to that specific stimulus, the preview also disappeared. If overlappers are, in principle, able to process this stimulus at any time in parallel with the performance of the relevant task, they should use it whenever it is presented.

## Experiment 2

Experiment 2 had two purposes. First, I tested whether the overlapping processor’s use of the preview depended on the particular position at which the preview was presented. Specifically, I tested whether the brief display of a preview was used when it was presented solely at the first, at the second, or at the third position of a TSWP sequence. The expected mean FSR per processing mode in each condition is illustrated in Fig. 6. Since there were no significant differences in overlappers’ FSRs between the conditions with different preview lengths in Experiment 1, it is conceivable that the preview could, in principle, be used at all three positions. However, two possibilities seem more likely. One possibility is that overlappers process the preview as soon as it is presented, that is, preferably on the first trial or, at the latest, on the second trial of a sequence. Such a processing order might be favored if participants experience the preview as rather salient and face difficulties in delaying its processing to a later point in the sequence. In this sense, it might be more convenient for these individuals to commit to processing the preview immediately and to store it until the information becomes beneficial for responding after the next task switch. Alternatively, overlappers may prefer to process the preview in parallel at the end of the sequence. Perhaps only after they have completed most of the processing of the currently relevant task sequence do they tend to engage in parallel processing as part of their preparation for task switching. In this case, increased use of the preview in the second and especially in the third trial of a sequence seems likely, as using the preview closer to the task switch would reduce working memory demands and potentially reduce task interference effects earlier in the sequence of the currently relevant task.

The second purpose of Experiment 2 was to test whether the slight increase in FSRs for serials and semi-overlappers found in Experiment 1 could be replicated. As in Experiment 1, Experiment 2 includes conditions in which the preview is systematically omitted (i.e., replaced by a \$  sign), which may lead to lower levels of task interference for these individuals, thus, fostering preview usage.

**Fig. 7:**
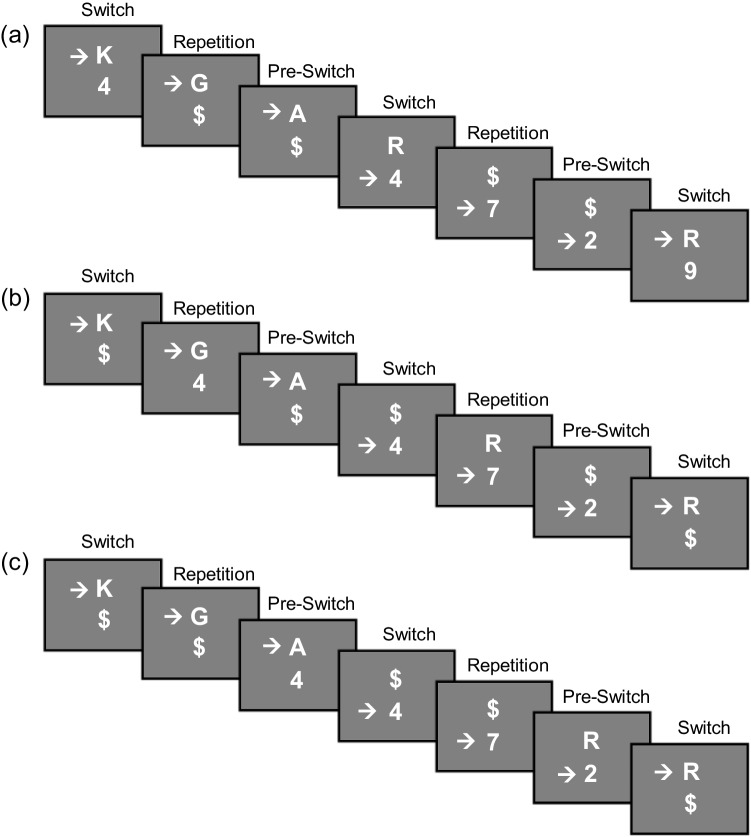
Task switching with preview conditions with discrete preview positions. *Note*. Sequence (**a**) shows the *early-preview* condition, where the preview is shown only in the first trial of a sequence in the task switching with preview (TSWP) paradigm. Sequence (**b**) shows the preview only in the second trial of a sequence in the TSWP paradigm, that is, the *middle-preview* condition. Sequence (**c**) shows the *late-preview* condition, where the preview is displayed only in the third trial of a sequence, which essentially corresponds to the *short-preview* condition of Experiment 1

### Method

#### Transparency and openness

Data were analyzed with the same software as reported in Experiment 1. The data obtained in Experiment 2 can be found in the same repository as that of Experiment 1.

#### Participants

A new sample of $n = 48$ volunteers was recruited via Prolific and was tested online (2021). The recruitment approach with respect to exclusion criteria was kept the same as in Experiment 1. All participants (16 female, 32 male; between 18 and 35 years, $M_{age} = 26$, 41 right-handed, six left-handed, one ambidextrous) were naïve regarding the hypotheses of this experiment, provided written informed consent before the experiment, and reported normal or corrected-to-normal vision. Participants received 8€  for participating and a monetary bonus of up to 4€  for the sum of correct answers within the TSWP. As in Experiment 1, no complete datasets had to be excluded, as all participants met the criteria of having less than 20% errors in any block of any condition and providing a sufficient number of trials (i.e., at least 40 trials per task and condition).

#### Tasks and stimuli

The tasks and stimuli were identical to Experiment 1, except that the preview was always presented just for one trial (see Fig. 7). Thereafter, the preview stimulus vanished and was replaced by a \$  sign. More precisely, an *early-preview* condition, a *middle-preview* condition, and a *late-preview* condition were introduced. The *early-preview* condition corresponded to the *full-preview* condition in its onset of the preview with the first trial of a sequence, but differed as the preview stimulus was replaced by the \$  sign in the second and third trial. Similarly, the *middle-preview* condition corresponded to the *medium-preview* condition in its onset of the preview with the second trial of a sequence, but differed as the preview stimulus was replaced by the \$  sign in the third trial. The *late-preview* condition exactly matched the *short-preview* condition as the \$  sign was shown in the first and second trial of a sequence, followed by the preview stimulus in the third trial.

#### Procedure

The procedure was largely identical to that of Experiment 1, with the main difference being that Experiment 2 included four conditions. In addition to the three experimental conditions (*early-preview*, *middle-preview*, and *late-preview*) needed to test the effect of preview position, the *full-preview* condition was retained to serve as a baseline for assessing participants’ preferred processing mode under a standard, unrestricted preview. In both the practice and experimental phases, all participants started with the *full-preview* condition. The subsequent experimental conditions were fully counterbalanced across participants using a Williams design (balanced Latin square) as in Experiment 1. Again, the order of conditions in the experimental phase matched the order experienced by each participant during the practice phase. The structure and duration of the single-task and task-switching blocks (e.g., 30 s, 60 s, or 120 s) were identical to those used in Experiment 1, as was the average overall duration of the experiment.

#### Design and analysis

The design and data analyses were similar to those of Experiment 1 with the mean FSR in the *full-preview* condition (i.e., the standard condition in previous studies) being the basis upon which participants were classified as overlapping, semi-overlapping, and serial processors, respectively. As in Experiment 1, this differentiation then served as a between-subject variable. Mean FSR of all four preview-position conditions was submitted to an ANOVA with preview-position (full vs. early vs. middle vs. late) as a repeated measure and processing mode (overlappers vs. semi-overlappers vs. serials) as a between-participants variable.

In Experiment 2, a total of 5.05% ($SD=1.18\%$) trials were discarded in the *full-preview* condition, 4.77% ($SD=0.83\%$) trials in the *early-preview* condition, 4.89% ($SD=0.83\%$) trials in the *middle-preview* condition, and 4.95% ($SD=0.75\%$) trials in the *late-preview* condition. On average, participants performed 175 trials in single-task blocks and 316 trials in task-switching blocks in the full-preview condition, 180 trials in single-task blocks and 327 trials in task-switching blocks in the early-preview condition, 182 trials in single-task blocks and 323 trials in task-switching blocks in the middle-preview condition, and 180 trials in single-task blocks and 343 trials in task-switching blocks in the late-preview condition (see Appendix A for more details).

### Results

Similar to Experiment 1, the mean PEs were overall low in Experiment 2. This was the case for the PEs in the single-task blocks in the *full-preview* condition, $M_{fullP} = 4.7$ and $SD_{fullP} = 3.1$, as for the *early-preview* condition, $M_{earlyP} = 4.7$ and $SD_{earlyP} = 3.2$, for the *middle-preview* condition, $M_{midP} = 5.3$ and $SD_{midP} = 3.5$, and for the *late-preview* condition, $M_{lateP} = 5.4$ and $SD_{lateP} = 3.6$. The PEs in the task-switching blocks were even slightly lower in the *full-preview* condition, $M_{fullP} = 3$ and $SD_{fullP} = 2.5$, in the *early-preview* condition, $M_{earlyP} = 3.1$ and $SD_{earlyP} = 2.3$, in the *middle-preview* condition, $M_{midP} = 3.5$ and $SD_{midP} = 3$, and in the *late-preview* condition, $M_{lateP} = 3.6$ and $SD_{lateP} = 2.8$. Although overall PEs were again relatively low, additional analyses were conducted to examine potential speed-accuracy trade-offs for each experimental condition and processing mode as in Experiment 1 (see Appendix B for details). These yielded again a significant main effect of trial type, and this time also of condition, but again with small absolute PE differences of about $1.2\%$ to $2.0\%$ for trial type and $0\%$ to $0.6\%$ for condition. Again, there were no processing modes or interaction effects. Therefore, the small PE differences are unlikely to fully account for the significant RT and FSR differences reported below.

#### Processing modes and RTs

Based on the distribution of FSR obtained in the *full-preview* condition of Experiment 2 (i.e., the standard condition in the TSWP paradigm), 15 participants were classified as overlappers, 7 as semi-overlappers, and 26 as serials. To take account of the natural differences in the sizes of the subgroups, I again used robust implementations of heteroscedastic ANOVAs based on trimmed means for all statistical comparisons involving the between-group factor.

**Fig. 8:**
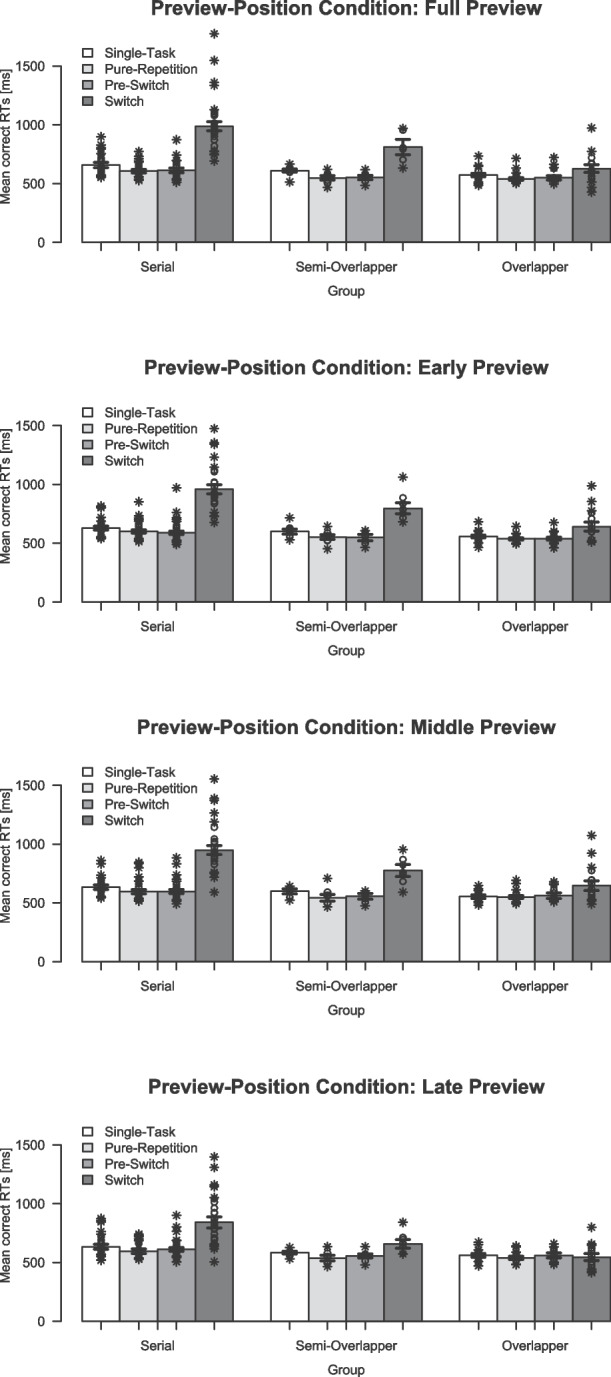
Mean response times ($M_t$) per trial type for each processing mode in each preview-position condition. *Note*. Depicted are the trimmed mean response times (RTs, in milliseconds) as a function of processor groups and preview-position condition. *Error bars* indicate ± one standard error calculated separately for each Trial Type in each Group $\times$ Preview-Position Condition. *Circles*/*crosses* represent individual data included/excluded in the calculation of trimmed means (20% criterion)

Trimmed means and individual RTs per trial type for each processing mode in each preview-position condition are visualized in Fig. 8. Similar to Experiment 1, RTs in the standard *full-preview* condition showed a typical pattern with short RTs (i.e., 539 ms to 659 ms) in single-task, pure-repetition, and pre-switch trials, but quite varying switch RTs across the processor subgroups. That is, the switch RTs of the *serial processor* were greatly prolonged by about $\hat{\Delta } = 361$ ms compared to the negligible prolongation of $\hat{\Delta } = 73$ ms of the *overlapping processor*. Again, the switch RTs produced by *semi-overlapping processor* were in between these extremes, that is, they were only moderately prolonged by $\hat{\Delta } = 240$ ms. Across the three preview-position conditions, the processor subgroups showed a similar pattern with respect to comparable RTs in single-task, pure-repetition, and pre-switch trials (i.e., 536 ms to 633 ms), but a high variability in the prolongation of switch RTs compared to the other trial types (i.e., from about $\hat{\Delta } = -7$ ms for overlappers in the *late-preview* condition to about $\hat{\Delta } = 353$ ms for serials in the *early-preview* condition). Overall, this pattern resulted in a main effect of trial type, $F(3, 135) = 138.27$, $p <.001$, $\eta _p^2 =.75$, as well as a main effect of processing mode in the robust ANOVA, $F(2,16) = 8.24$, $p =.003$, $KMS = 0.38$, and an interaction between processing mode $\times$ trial type, $F(6,15) = 9.09$, $p <.001$, $AKP = 1.08$.

**Fig. 9:**
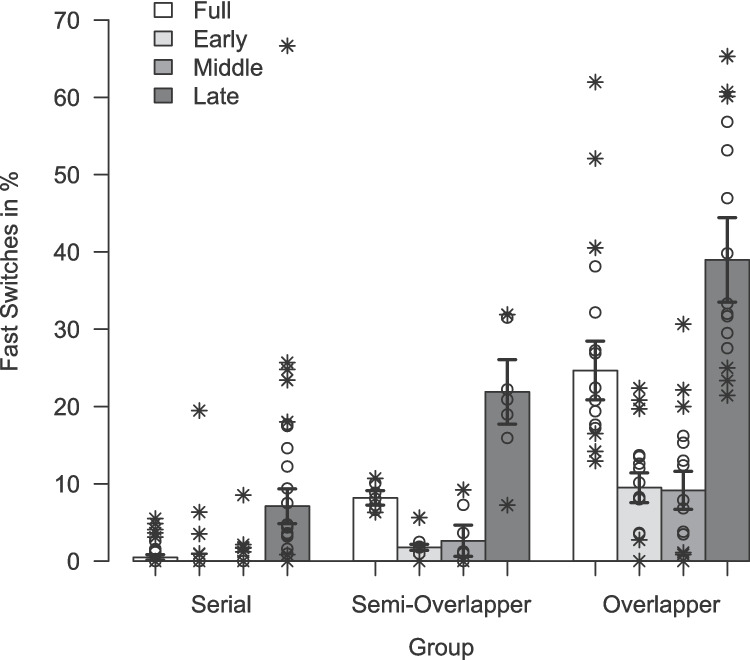
Mean fast switches (in %) for Experiment 2. *Note*. Depicted are the average percentage fast switches as a function of processor groups and preview-position condition (calculated on trimmed means).* Error bars *indicate ± one standard error calculated on trimmed means, separately for each Group $\times$ Preview-Position Condition. *Circles*/*crosses* represent individual data included/excluded in the calculation of trimmed means (20% criterion)

Interestingly, also the main effect of preview position was significant, $F(2, 90) = 23.40$, $p <.001$, $\eta _p^2 =.34$ with overall faster responses in the *late-preview* condition, $M_a = 632$ ms, than in the *early-preview*, $M_a = 657$ ms, and *middle-preview*, $M_a = 662$ ms, condition. These differences resulted from a divergent pattern in the switch RTs as compared to the other trial types, which is also reflected in an interaction between preview-position condition and trial type, $F(6, 270) = 43.48$, $p <.001$, $\eta _p^2 =.49$. Regarding the factor processing mode, the *overlapping processors* showed rather similar switch RTs in the *early-preview* and *middle-preview* conditions compared to the *full-preview* condition (i.e., slight increases of $\hat{\Delta } = 13$ ms and $\hat{\Delta } = 19$ ms, respectively). Remarkably, their switch RTs were even shorter in the *late-preview* condition than in the *full-preview* condition (i.e., $\hat{\Delta } = 81$ ms decrease), reduced to the level of the other trial types. A similar picture, albeit weaker, emerged for serial and semi-overlapping processors. Although their switch RTs remained consistently longer than all other trial types in all conditions, they also showed a substantial reduction in switch RTs in the *late-preview* condition compared to all other preview-position conditions. But, neither the two-way interaction preview position $\times$ processing mode nor the three-way interaction preview position $\times$ processing mode $\times$ trial type reached statistical significance for the RTs (all $p >.308$).

#### Fast switch rates

The mean FSR ($M_t$), reflecting the extent to which participants preprocessed the preview in an overlapping mode, is depicted per processor group in each condition in Fig. 9. The corresponding robust ANOVA yielded a main effect of processor group reflecting the general differences in mean FSR between processor groups resulting from the categorization approach, $F(2,12.74) = 25.59$, $p <.001$, $KMS = 0.72$. Accordingly, the FSR were generally higher for overlapper ($19.4\%$) than for semi-overlapper ($6.5\%$), and serial processors ($0.8\%$). This typical stepwise increase in FSR resulting from the categorization approach is also clearly evident in the standard TSWP condition, that is, the *full-preview* condition, mirroring the pattern of results observed in the switch RTs.

The robust ANOVA further revealed a significant main effect of preview-position condition, $F(3,12.27) = 32.90$, $p <.001$, $AKP = 1.27$, with an overall considerably higher FSR in the *late-preview* condition ($M_t = 17.9\%$) than in the *early-preview* and *middle-preview* conditions ($M_t = 1.8\%$ and $M_t = 1.6\%$). Accordingly, the interaction of preview-position condition and processing mode was statistically significant as well, $F(6,12.77) = 9.87$, $p <.001$, $AKP = 1.38$. The three processing groups showed a distinct pattern in the preview-position conditions: For overlapper, FSR were lower in the *early-preview* and *middle-preview* conditions (9.5% and 9.2%), and considerably increased in the *late-preview* condition ($39.0\%$) compared to the *full-preview* condition (24.7%). This difference was less pronounced, but also significant, for serial and semi-overlapping processors: For serial processors, FSRs were similarly low in the *early-preview* and *middle-preview* conditions ($<0.1\%$ and $<0.1\%$) as in the *full-preview* condition (0.5%). Semi-overlapper showed even slightly lower FSRs in the *early-preview* and *middle-preview* conditions (1.8% and 2.6%) compared to the *full-preview* condition (8.2%). Remarkably, both serial and semi-overlapping processor groups showed on average higher FSRs in the *late-preview* condition (7.1% and 21.9%) compared to the respective values in the *full-preview* condition.

### Discussion

The primary goal of Experiment 2 was to examine whether the position of the preview in the TSWP paradigm influences its use, and thereby the occurrence of overlapping processing. To this end, three conditions were introduced in addition to the previously used *full-preview* condition (Brüning & Manzey, 2018; Brüning et al., 2021, 2022; Reissland & Manzey, 2016). In the *full-preview* condition, the preview is available throughout the entire sequence. In contrast, it appears only in the first trial of a sequence in the *early-preview* condition, in the second trial in the *middle-preview* condition, and in the third trial – immediately before the switch – in the *late-preview* condition. If preview position is indeed the critical factor determining its use by overlapping processors, it should be predominantly used in one of the preview-position conditions – most likely either at the beginning of a sequence (with the task switch) or at its end (just before the next switch).

For the *full-preview* condition, the FSR pattern and the resulting numbers of serials, semi-overlappers, and overlappers obtained are similar to previous results (Brüning & Manzey, 2018; Brüning et al., 2021, 2022; Reissland & Manzey, 2016). The main effect of processing mode shows that these differences persist at a general level, across preview-position conditions. More interestingly, however, in Experiment 2 there was an interaction effect between the preview-position condition and the processing mode on the FSRs. The results for overlapping processors are straightforward: the preview is mainly used for overlapping processing right before a switch. This is about four times more common than in the first or second part of a sequence. Nonetheless, most overlapping processors still show some instances of overlapping processing in the *early-preview* and *middle-preview* conditions, which indicates that they are still able to use the preview for overlapping processing under those circumstances. Overall, the results show that preview position critically affects the time course of preprocessing an upcoming task, and that overlapping processing can occur even when the preview is available only shortly before the switch – requiring rapid task preparation.

Besides, Experiment 2 allowed me to test whether the increase in FSR for serial and semi-overlapping processors could be replicated when there was no preview presentation at the beginning of a sequence and thus a lowered risk of task interference. Remarkably, although serial and semi-overlapping processors show considerably lower FSRs overall than overlapping processors, they exhibited a similar pattern across the *early-preview*, *middle-preview*, and *late-preview* conditions. In particular, both groups show almost no incidences of overlapping processing in the *early-preview* and *middle-preview* conditions. In the *late-preview* condition, however, the semi-overlappers adopt an overlapping processing mode to a degree that resembles the overlapping processors’ mode in the standard *full-preview* condition. Moreover, when the preview is presented just directly before the switch, even serial processors begin to show significantly more incidences of an overlapping processing mode. Taken together, this pattern represents a conceptual replication of the finding that the preview at the beginning of a sequence does not lead to overlapping processing in serial and semi-overlapping processors, whereas a preview presented closer to the switch can promote it. In this vein, the pattern of results further supports the notion that the preview might to some degree rather interfere with the processing of the currently relevant task for individuals who tend to process serially. However, if preview information is provided not at the beginning of a sequence, but close to the upcoming task switch, the risk of task interference may be significantly reduced. This will be further discussed in the following section.

## General discussion

The present study investigated whether the use of a preview to preprocess a second task in a task-switching situation depends on the length of the preview or its position. In doing so, the study aims to shed light on when exactly parallel processing occurs in multitasking situations characterized by predictably alternating task switches. To this end, blockwise varying conditions of different preview lengths (Experiment 1) versus different concrete positions at which the preview is displayed (Experiment 2) were introduced. Specifically, Experiment 1 compared preview conditions of different durations: short, medium, and long (i.e., one vs. two vs. three trials of preview). I assumed that a longer preview presentation would increase the likelihood of overlapping processing. Experiment 2 examined the use of the preview when it was presented on either the first, second, or third trial of a sequence. For Experiment 2, I assumed that overlapping processing is facilitated either at an early onset of the preview, when the new information of the preview stimulus is salient, or at a later onset, when processing the preview is less demanding on working memory.

### Summary of the results and theoretical interpretation

With respect to the main question of the current study as to when exactly a provided preview is used, the results of the presented experiments are straightforward. First of all, the length of the preview seems to be of less relevance. It is therefore evident that the accumulation of information does not occur at a slow and steady pace until a certain threshold is reached; rather, it is a rapid process. More importantly, the exact position of the preview information is an essential aspect. The presented data suggest that when all information about two tasks is always available (like the *full-preview* condition), individuals who preprocess an upcoming task in parallel predominantly use the information provided by the preview directly before the next switch occurs. This is especially evident in the *late-preview* condition in Experiment 2, where these participants show a decisive increase in their overlapped processing indicated by increased FSRs. As much as the use of preview information to prepare the task switch is facilitated by such a late onset, it is rather hindered by an early onset of preview, as indicated by reduced FSRs in Experiment 2. This distinction was rather unexpected and has several implications.

Regarding the preprocessing of the upcoming task stimulus, one conceivable explanation for the preview benefit in the TSWP paradigm is a passive, automatic activation of the representation of the upcoming stimulus. Once the preview becomes available, this activation may occur inevitably, such that the stimulus is processed automatically, resulting in faster switch responses under favorable conditions of low task interference. Characterizing the use of the preview as such a passive process requires that stimuli from both tasks are within the focus of attention. When the preview appears at the first position of a sequence, both the cue and the stimulus from the last task performed – now serving as the preview – change. Similarly, when the preview appears in the second position, there is a change not only in the stimulus of the performed task, but also in the previewed stimulus. In both cases, automatic processing due to the salient onset of the preview stimulus remains theoretically plausible due to the close spatial proximity of the stimuli and the likely width of the attentional focus (see, e.g., Ellenbogen & Meiran, 2011; Lehle & Hübner, 2009, for similar effects of spatially close stimuli in related dual-task settings).

However, several observations argue against a purely passive mechanism. For example, it could be argued that either the shift of the cue (first position) or changes in the currently relevant task (second position) draw attention more strongly to the relevant task and, crucially, away from the preview stimulus. In this sense, using the preview may indeed require a shift of attention and thus involve top-down processing. More importantly, if the processing of the preview were purely automatic, it should take effect immediately upon the onset of the preview stimulus, regardless of whether the preview appears in the first, second, or third position of the sequence-yet the magnitude of the benefit clearly depended on the preview position in the present study. One could further argue that in case of an earlier preview onset, the activation of the preview stimulus does simply not last long enough. That is, it decays already before the task switch and can thus not result in faster switch responses (or only in rare occasions). While this interpretation cannot be completely ruled out, it seems rather unlikely given that the strength of preactivated response information varies with its relevance. In particular, the more relevant a task is, the stronger and longer its associated information remains in the system (Koob, Ulrich, & Janczyk, 2023; Mittelstädt, Mackenzie, Koob, & Janczyk, 2023). For example, Mittelstädt et al. (2023) varied the relevance of typically only distracting information in conflict tasks (e.g., responding to flankers in an Eriksen flanker task) and showed that with increasing relevance, the activation of this additional information tended to persist against decay. Similarly, Miller and Tang (2021) showed that responses to a second task were substantially faster in blocks where the task became more relevant, consistent with the idea that the second task actually reached a higher activation. Accordingly, since the preview in the TSWP paradigm also increases in relevance over time (i.e., when the task switch gets closer in time), its relevance and thus its activation is more likely to increase rather than decrease. The assumption of an automatic process underlying the differential effect of preview position is also contradicted by previous findings on interference effects in the TSWP (Brüning & Manzey, 2018). This study compared the effects of high versus low risk of task interference during preview use. Participants performed either univalent or bivalent tasks. The former consisted of visually distinct tasks, while the latter consisted of the same stimulus material. If the processing of the preview were to be a passive, automatic process, a preview involving a high risk of interference would have been expected to impair the performance of the currently relevant task. Notably, the increase in interference only affected the switch performance, whereas there was no immediate effect on the performance of the currently relevant task.

Accordingly, the simplest and most obvious explanation would be that the processing takes place actively, that is, it is a top-down process. But if so, why does it most often occur directly before the switch, rather than as soon as the preview information becomes available? After all, in line with the task-set reconfiguration account by Rogers and Monsell (1995), both the endogenous as well as the exogenous component should, in principle, be completed before a task switch in TSWP as all information is given and ample preparation time is warranted. Moreover, the pattern of results obtained in Experiment 2 suggests that it is not impossible for overlapping processors to use the preview when it is presented early. Two factors could account for this shift in probability in favor of late preview use. First, processing the preview at the beginning of the sequence would entail a higher demand on working memory. In contrast, the later participants process the preview, the fewer mental operations are still necessary to be performed in the concurrently relevant task. For example, if the participant manages to time the processing of the preview with encoding the preview stimulus in the pre-switch trial, they only need to perform one more concurrent mental operation on the currently relevant task. If the processing of the preview is timed perfectly with the motor response in the pre-switch trial, even no further mental operations would be necessary before the participant can turn to the task switch stimulus. In this vein, a later usage of the preview just when it becomes relevant, is also the most sensible approach to optimize performance. The fact that overlapping processors in fact use the preview not solely at the very last trial of a sequence may be due to the fact that they tend to have a higher working memory capacity (Brüning & Manzey, 2018), allowing for some overlap in mental operations.

### Speculations on a preview interference effect

The second factor that could, in principle, account for a late preview usage is task interference. That is, while we are usually inclined to view the preview as helpful information that allows for advanced preparation of the response in the next task switch, it could actually have detrimental effects by potentially interfering with the processing of the currently relevant task stimulus. This becomes particularly apparent when considering task interference accounts such as “task-set inertia” (i.e., passive interference due to passive task-set decay, Allport et al., 1994) or “backward inhibition effects” that can occur when a task set that was once inhibited for a previous switch must now become relevant for processing the upcoming switch trial (Mayr & Keele, 2000). Following these accounts, potential task interference from a second task set (i.e., in this case from the preview of the other task) is high directly after a task switch and fades thereafter. In the case of the TSWP paradigm, this type of task interference might be somewhat increased or might last even a little longer. After a task switch to Task B (A) the previously performed Task A (B) does not simply vanish, but in the classical full-preview TSWP condition, the next stimulus of Task A (B) is directly presented as a preview until the subsequent task switch back to Task A (B). This could potentially hamper the deactivation of the no longer relevant Task A (B) when participants have to work on the currently relevant Task B (A) in the beginning of a sequence. However, avoiding the interference of the previous Task A (B) may be more important in the beginning of a sequence than when the (next) performance of the task gets closer in time. The timing of these various processes involved (i.e., shifting between tasks, *and* handling task interference) can be challenging. If the preview information is presented late in the sequence, it may support their coordination and become therefore particularly effective.

The notion that an early display of the preview might rather cause task interference is further supported by the data pattern of serial processors. While the serial processor’s performance has never benefited from a full preview in previous studies (e.g., Brüning & Manzey, 2018; Brüning et al., 2021; 2022), this is the first study to show that they can benefit to some extent from the preview, provided it is displayed late in the sequence. This implicitly suggests that an early preview start, as previously used, was more of a hindrance for serial processors. While the current findings cannot provide an empirical answer as to why serial processors show at least some instances of overlapping processing in a late-preview but not in a full-preview condition, I might provide some speculations on the matter. Given the current state of the literature, this distinction may reflect that serial processors are much more likely to be in a task-shielding mode than overlapping processors. That is, they actively shield the processing of the currently relevant task stimuli from any potentially interfering processing of the preview stimulus (see e.g., Dreisbach & Wenke, 2011; Fischer et al., 2018; Zwosta, Hommel, Goschke, & Fischer, 2013, for accounts of flexible and gradual task-shielding). In the TSWP paradigm, serial processors can, in principle, focus just on the currently relevant task and shield their information processing from other influences to avoid potential causes of task interference (i.e., the preview). This may seem like a suboptimal solution, since it prevents serial processors from benefiting from the preview, even though it is always available. However, it may actually be a more adaptive solution for them. Indeed, it has been recently reported that serial processors may be more susceptible to task interference in general than overlapping processors (Brüning et al., 2022). This susceptibility was particularly evident in situations of high information density or when multiple tasks had to be processed simultaneously in a dual-task. Those participants who preferred a serial processing mode in the TSWP paradigm showed higher inter-task interference.

### Limitations and future avenues

One potential limitation is that Experiment 1 was conducted in the laboratory, whereas Experiment 2 was conducted online. Although stimulus timing, input devices, and participant demographics (e.g., age or tech familiarity) were comparable across both samples, the online testing environment naturally offers less control. This may have introduced more variability in environmental distractions or participant focus. While I do not believe this explains the key differences between conditions in Experiment 2 – for example, similar patterns emerged for serial and semi-overlapping processors in both experiments – it cannot be entirely ruled out that such factors contributed to a greater reliance on the preview when it was provided directly before a switch among overlapping processors. I note this possibility with caution, as it may have slightly amplified the observed shift in preview use on the last trial in Experiment 2. However, it seems highly unlikely that a laboratory experiment would show the opposite pattern of results.

The considerations outlined in the General discussion focus on the mechanisms behind the preferred use of late preview information and the relationship between task processing modes, task interference susceptibility, and working memory capacity. However, further research is needed to clarify these relationships more clearly. Furthermore, although we now know when the preview is preferentially used, it remains unclear how exactly the preview is used to accelerate task switching beyond the mere encoding of the preview stimulus. This could involve both the anticipatory initiation of the task-set reconfiguration or even the response selection and the preparation of the subsequent switch response. Future studies could address these open questions through the use of more fine-grained methodological approaches. One promising avenue would be the application of diffusion models (Ratcliff, 1978), which allow the decomposition of observed RTs and accuracies into latent parameters reflecting distinct cognitive processes, such as evidence accumulation, nondecision time, and response caution. Such models have already been fruitfully applied to task-switching paradigms (e.g., Schmitz & Voss, 2012) and, more recently, have been adapted to account for dual-task processing (Koob et al., 2023). In the context of the current paradigm, comparing model parameters across participant subgroups could help identify how individuals who spontaneously engage in preview-based preparation differ from those who do not –particularly with respect to anticipatory processes indexed by reductions in nondecision time (see Imburgio & Orr, 2021, for similar findings in a voluntary task switching paradigm).

Beyond these more fundamental mechanisms, the efficient use of preview information may be of interest in more applied research settings, such as those addressed by human factors research. Consider the presentation of information in more complex environments where frequent task switching is required, such as when a pilot monitors multiple displays in an aircraft, or an operator monitors multiple displays in a control room. In such environments, displays for tasks that need to be switched between should also be displayed in close proximity to each other, so that all displays relevant to a particular (sub-)task can be easily previewed and quickly switched between. In fact, Wickens and Carswell (1995), who proposed the Proximity Compatibility Principle, noted that such a grouping of displays should be considered in the design of technology. This principle states that spatial grouping of displays is advisable because, for example, it reduces the time required for saccades from one display to the next and, on a cognitive level, facilitates the integration of information across displays. The current findings not only support this notion but also extend the reasoning by suggesting that grouping displays also facilitates previewing the surrounding displays in parallel to enable efficient switching between tasks, especially in work environments where multitasking is essential.

### Conclusion

The present study extends previous work on the classical TSWP paradigm by employing conditions of varying length and position of the preview information provided to investigate when such information is used to prepare for a second task in a task-switching scenario characterized by frequent periodic switches. Indeed, the results show that preview information is mainly used just before a task switch. This was observed primarily in individuals who prefer (semi-)overlapping processing, but surprisingly and to a much lesser extent also in serial processors. Overall, it can be concluded that the ability to use preview information for task preparation is enhanced when preview information is provided at relevant moments, that is, shortly before a switch. Exactly how the time course of such parallel processing relates to the concepts of working memory load and task interference remains a matter of speculation, but promises to be a worthwhile future avenue in this area.

## Figures and Tables

**Fig. 10:**
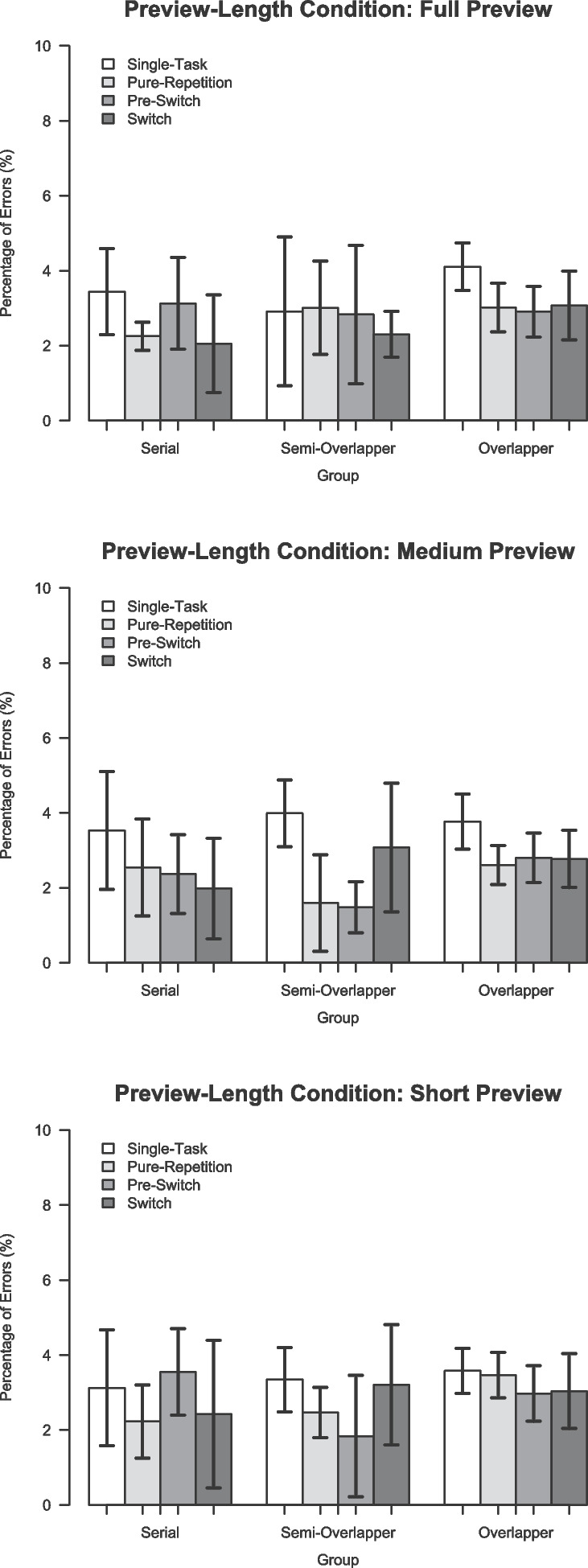
Mean percentage errors for experiment 1. *Note*. Mean percentage errors per trial type for each processing mode in each preview-length condition. *Error bars* indicate ± one standard error

**Fig. 11:**
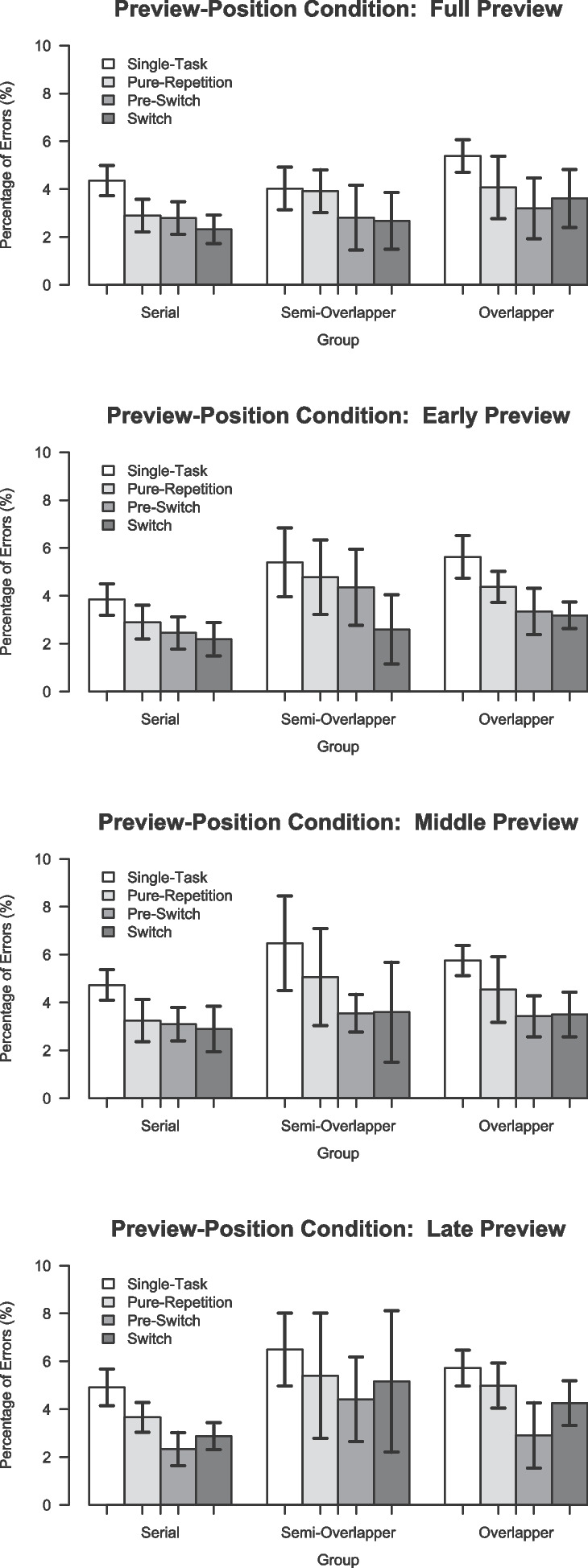
Mean percentage errors for Experiment 2. *Note*. Mean percentage errors per trial type for each processing mode in each preview-position condition. *Error bars* indicate ± one standard error

## Data Availability

The datasets generated and analyzed during the current study are available in the OSF repository, https://osf.io/ry3pz/?view_only=aa21a94eb9e64c8a85c1d735b225148a. The custom code for analyzing the data is available from the corresponding author on reasonable request.

## Appendix A

This appendix provides details on the number of analyzed trials in both experiments.

### A.1 Number of trials analyzed in Experiment 1

**Table 1:** Number of trials analyzed in single- and task-switching blocks across preview-length conditions

Conditions	$M_{ST}$	$Min_{ST}$	$Max_{ST}$	$SD_{ST}$	$M_{TS}$	$Min_{TS}$	$Max_{TS}$	$SD_{TS}$
Full Preview	172	126	218	21.8	317	216	432	57.8
Medium Preview	174	134	222	20.8	322	211	422	53.8
Short Preview	170	124	207	22.0	319	221	445	51.6

Note. *M* = mean, *Min* = minimum, *Max* = maximum, *SD* = standard deviation; *ST* = single-task, *TS* = task-switching

### A.2 Number of trials analyzed in Experiment 2

**Table 2:** Number of trials analyzed in single- and task-switching blocks across preview-position conditions

Conditions	$M_{ST}$	$Min_{ST}$	$Max_{ST}$	$SD_{ST}$	$M_{TS}$	$Min_{TS}$	$Max_{TS}$	$SD_{TS}$
Full Preview	175	108	237	27.2	316	157	458	65.2
Early Preview	180	115	250	26.9	327	186	442	60.4
Middle Preview	182	121	238	26.6	323	186	446	62.0
Late Preview	180	118	240	26.9	343	200	463	65.0

Note. *M* = mean, *Min* = minimum, *Max* = maximum, *SD* = standard deviation; *ST* = single-task, *TS* = task-switching

## Appendix B

This appendix provides details on additional analyses of PEs in both experiments, which were conducted to examine potential speed–accuracy trade-offs for each experimental condition and processing mode. Condition-wise PEs are presented in Fig. 10 for Experiment 1 and in Fig. 11 for Experiment 2.

I performed ANOVAs on PEs using the same procedure as for the RT analyses. A main effect of trial type was observed in both experiments. In Experiment 1, PEs were higher in the single-task condition (3.62%) compared to the other trial types (ranging between $2.72\%$ and $2.77\%$), $F(3, 135) = 4.24$, $p =.013$, $\eta _p^2 =.09$. A similar pattern emerged in Experiment 2, with PEs again higher in the single-task condition ($4.99\%$) relative to the remaining trial types (ranging between 3.0% and 3.82%), $F(3, 135) = 33.44$, $p <.001$, $\eta _p^2 =.43$. Additionally, in Experiment 2, a main effect of condition emerged, $F(3, 135) = 5.86$, $p =.002$, $\eta _p^2 =.12$, with the middle (3.92%) and late (4.04%) conditions showing slightly elevated PEs compared to the full ($3.44\%$) and early ($3.46\%$) conditions. This reflects a relatively small absolute difference of about 0.5%.

To account for potential group differences, I also conducted robust ANOVAs on trimmed means of PEs for both experiments. In Experiment 1, overlapping processors (2.83%) made slightly more errors than serial processors (2.13%) – by $0.7\%$. In Experiment 2, again overlapping processors (3.64%) made slightly more errors than serial processors ($2.79\%$) – by 0.85%. However, neither the main effects of processing mode nor any interaction effects reached significance (all $p >.117$).

## Footnotes

1 It is important to note that beyond the general process of shifting between tasks—which will be further specified later in the introduction—it remains unclear which specific processing stages can operate in parallel during overlapping processing. Such overlap may be limited to perceptual encoding of stimuli, or it may also extend to response activation, response selection, and the execution of motor programs. However, addressing this question lies beyond the scope of the present study.

2 It should be noted that fast switch rates are a continuous metric. Accordingly, a minority of participants may only occasionally show fast switches, which are typically too infrequent to indicate systematic use of the preview. Such participants are therefore referred to as *semi-overlapping processors*.

3 One of the participants reported a date of birth that indicated he was 89 years old. However, this must have been a typographical error that went unnoticed (i.e., 1929 instead of 1992), because it was clear from visual inspection that the participant was under that age. When reporting the age values, I omitted this participant’s age.

4 Note that this procedure is consistent with previous studies using the TSWP paradigm, where it was essential to calculate performance efficiency not only in terms of costs and benefits for each trial, but also as an overall task throughput (i.e., by comparing the number of dual- and single-task trials).
